# A Synthetic View on Momilactones and Related 9*β*-H Pimarane Skeleton Diterpenoids

**DOI:** 10.3389/fchem.2022.882404

**Published:** 2022-03-21

**Authors:** Yue Zhang, Mengran Li, Qichang Liu, Jian Huang, Yang Chen

**Affiliations:** State Key Laboratory Breeding Base of Green Pesticide and Agricultural Bioengineering, Key Laboratory of Green Pesticide and Agricultural Bioengineering, Ministry of Education, Research and Development Center for Fine Chemicals, Guizhou University, Guiyang, China

**Keywords:** 9*β*-H pimarane, skeleton, momilactones, allelochemical, diterpenoids

## Abstract

Allelochemicals are secondary metabolites produced from plants and used to prevent and control the invasion of other plants and microorganisms, with broad application prospects in crop protection. Structurally, momilactones belong to 9*β*-H pimarane diterpenoids, one of rice’s significant allelochemicals with anti-weeds and antibacterial activity. Rare studies have been reported with the synthesis challenges of the unique 9*β*-H pimarane skeleton. Hence, synthetic strategies of momilactones and related 9*β*-H pimarane skeleton are reviewed from 1984 to 2021.

## Introduction

**FIGURE 1:**
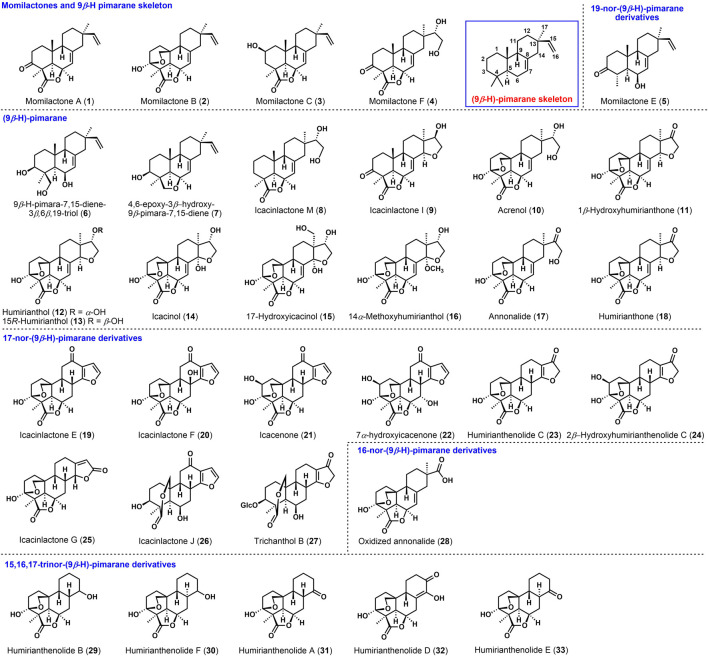
9*β*-H-Pimarane related diterpenoids and the 9*β*-H pimarane skeleton.

Modern genetic evidence and recent studies have shown that momilactones are among the most active allelochemicals (Lin et al., 2019) and play a key role in allelopathy and resistance induction in rice (Okada et al., 2016). In 1973, momilactone A (**1**) and momilactone B (**2**) were isolated from *Oryza sativa* L. by Kato (Kato et al., 1973), firstly identified as new growth inhibitors. They have significant bioactivities, including weeds elimination in paddy fields and antimicrobial activity, especially toward *Pyricularia oryzae* Cav. (Jiang et al., 2016). However, the natural content of momilactones could not meet further research needs. Synthetic approaches to yield these natural products seem to attract synthetic chemists (Mohan et al., 1996). Kato (Kato et al., 1977) determined the stereochemical configuration of momilactone A by X-ray single-crystal diffraction as 9*β*-H. Momilactone A has continuous chiral centers with a *trans-syn-cis* tricyclic skeleton named 9*β*-H pimaranes, as shown in Figure 1, characterized in the family compounds (Zhao et al., 2018). Moreover, the *trans-syn-cis* tricyclic ring and the stereochemistry at C-9 led to significant challenges in synthesizing these molecules. In the early stage (Deslongchamps and Germain, 1999), the construction of the 9*β-*H-pimarane skeleton commonly had drawn the attention of scientists devoted to the synthesis of momilactones and related diterpenoids.

9*β*-H-pimarane diterpenoids are featured with the *trans-syn-cis* tricyclic skeleton and *β*-configuration of the proton at C-9. Such studies have been reported to investigate their abundant biological activities (Xu et al., 2021). Figure 1 shows that the known (9*β*-H)-pimarane related diterpenoids can be classified into (9*β*-H)-pimarane (**1**–**4**, **6**–**18**), 16-*nor*-(9*β*-H)-pimarane (**28**), 17-*nor*-(9*β*-H)-pimarane (**19**–**27**), 19-*nor*-(9*β*-H)-pimarane (**5**), and 15,16,17-tri*nor*-(9*β*-H)-pimarane derivatives (**29**–**33**). Among the momilactone family, momilactones A and B were obtained from moss *Hypnum plumaeforme* by Nozaki (Nozaki et al., 2007). Momilactones C (**3**), F (**4**), and E (**5**) were found from the hulls (Liu et al., 2012), leaves, and roots of rice (Cho et al., 2015). Strictly speaking, momilactone E belongs to 19-*nor*-(9*β*-H)-pimarane, and momilactone D possesses the 9*β*-OH, which could not be classified as (9*β*-H)-pimarane. These natural products exhibited inhibition of weeds and antibacterial activities (Tsunakawa et al., 1976). Momilactone B had the most efficient, currently known bioactivity (Dayan et al., 2009).

For example, (9*β*-H)-pimaranes, 4,6-epoxy-3*β*-hydroxy-9*β*-pimara-7,15-diene (**7**), and 9*β*-H-pimara-7,15-diene-3*β*,6*β*,19-triol (**6**) (Horie et al., 2015) were isolated from the rice husks of *Oryza sativa* L. The anti-fungal activities on *Magnaporthe grisea* (Li et al., 2014) have been investigated. Icacinlactone M (**8**), 14*α-*methoxyhumirianthol (**16**), and annonalide (**17**) were found from *Icacina oliviformis* (Zhao et al., 2015a) for the first time (Sun et al., 2021). Besides (Graebner et al., 2000), humirianthol (**12**) (Li et al., 2020), icacinol (**14**), 17-hydroxyicacinol (**15**), 14*α*-methoxyhumirianthol (**16**), and annonalide (**17**) showed cytotoxic activities (Onakpa et al., 2014) toward human cancer cell lines. These compounds were also obtained from the tuber of *Icacina oliviformis* (Zhou et al., 2020). Cytotoxic humirianthone (**18**) and 15*R*-humirianthol (**13**) were found from the lianas in the Suriname rainforest (Adou et al., 2005). The 17-*nor*-(9*β*-H)-pimarane derivates (Zhao et al., 2015b), humirianthenolide C (**23**), 2*β*-hydroxyhumirianthenolide C (**24**), icacenone (**21**), 7*α*-hydroxyicacenone (**22**), and icacinlactone E-J (**19**, **20**, **25**, **26**) with cytotoxic activities (Guo et al., 2016) were isolated from the tubers of *Icacina trichantha* (Zhao et al., 2015a). 7*α*-Hydroxyicacenone (**22**), icacenone (**21**), and trichanthol B (**27**) (Xu et al., 2021) might also be considered for antimicrobial activities (On'Okoko et al., 1985). Humirianthenolides A, B, D, E, and F (**29**–**33**) were separated from the tuber of *Humirianthera rupestris*, known as the 15,16,17-tri*nor*-(9*β*-H)-pimarane derivates (Zoghbi et al., 1981). Oxidized annonalide (**28**) was identified as 16-*nor*-(9*β*-H)-pimarane derivates. Most of the above compounds exhibited biological activities such as plant growth inhibition, anti-fungal activity (Shen et al., 2020), and cytotoxicity (Zhou et al., 2020). Given the broad biological activities, the chemical syntheses of 9*β*-H-pimarane diterpenoids are significant, although there was only one total synthesis of (±)-momilactone A reported by Germain and Deslongchamps Germain and Deslongchamps (2002). This review covers the recent synthetic approaches to momilactones and related 9*β*-H-pimarane skeleton.

## Synthetic Studies Toward 9β-H Pimarane Skeleton Diterpenoids

A few synthetic strategies about 9*β*-H-pimarane skeleton molecules had been described for the challenging framework, especially the continuous chiral centers. It would be difficult to accomplish the *trans-syn-cis* tricyclic with stereoselectivity.

**SCHEME 1:**
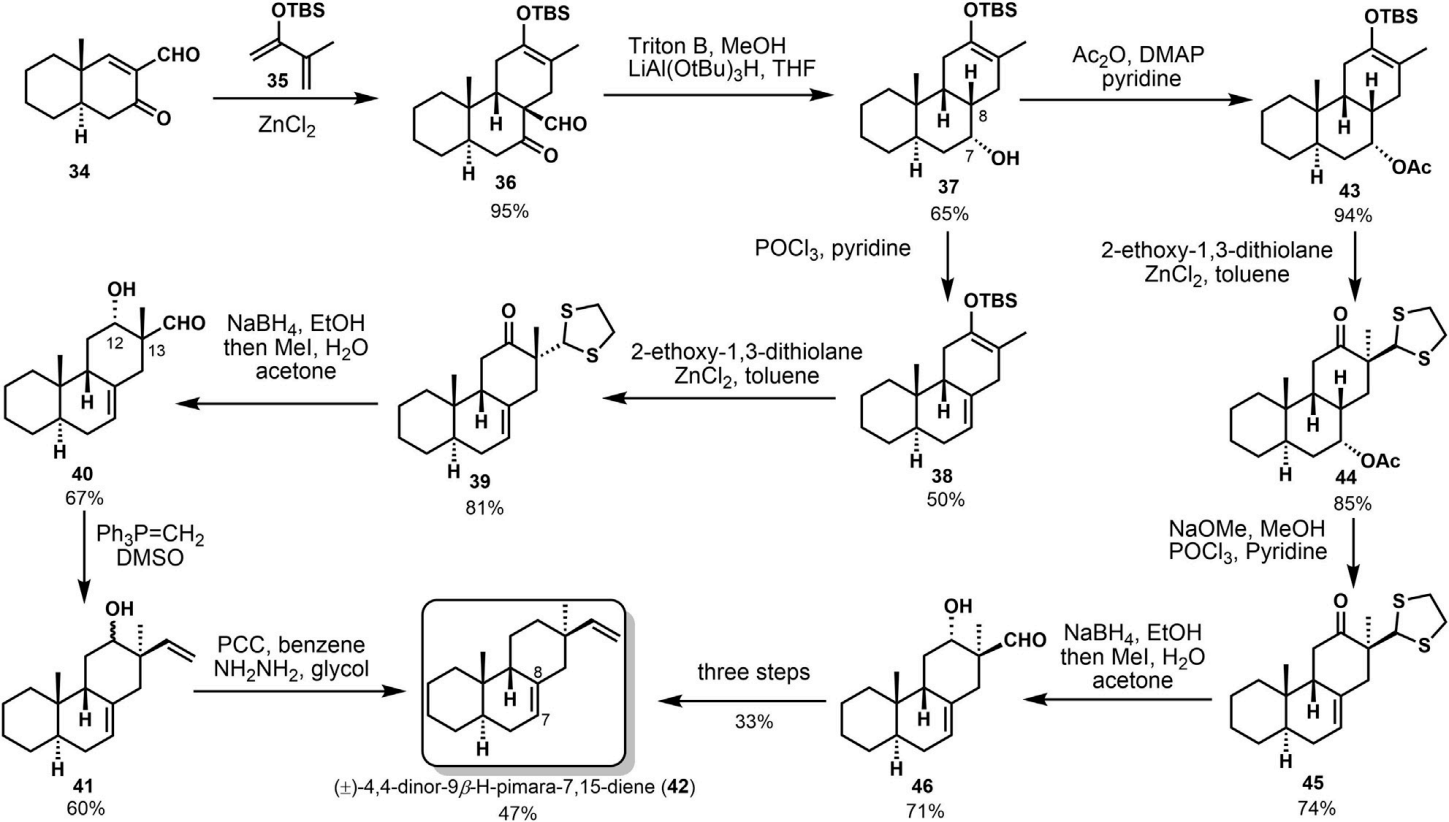
De Groot’s first synthesis of (±)-4,4-dinor-(9*β*-H)-pimara-7,15-diene in 1984.

In 1984, Sicherer-Roetman (Sicherer-Roetman et al., 1984) described the synthesis of model compound (±)-4,4-dinor-(9*β*-H)-pimara-7,15-diene **42**, possessing the *trans-syn-cis* skeleton and *α*-methyl and *β*-vinyl groups at C-13. The transannular Diels–Alder strategy had been used to construct the core tricyclic system, as shown in Scheme 1. Product **36** was obtained by the Diels–Alder reaction of ketone formaldehyde **34** and o-diolefin **35** under the catalysis of ZnCl_2_; the step provided that *cis*-adduct **36** was deformylated in the presence of triton B and then hydrogenated with LiAl(O*t*Bu)_3_H to obtain sole reduction product **37**. From this point on, compound **42** could be provided by two different strategies. First, compound **37** was dehydrated in POCl_3_ and pyridine to yield dienolsilane **38**. Then, dithioacetal **39** was obtained with 2-ethoxy-1,3-dithiolan, and *cis-β*-hydroxyaldehyde **40** was afforded by reduction and hydrolysis. They got *β*-vinyl product **41** through a Wittig reaction of compound **40**. Considerable epimerization occurred at C-12 and C-13, a handful of the *α*-vinyl product was detected. Finally, oxidation of **41** and Wolff–Kishner reduction of the carbonyl gave compound **42** at 47% yield. The second approach protected the hydroxyl group to afford acetyl ester **43**. Alkene intermediate **45** was afforded through the alkylation, hydrolyzation, and dehydration, followed by reduction and hydrolyzation. With compound **46** in hand, epimerization also occurred, resulting in a single *β*-vinyl product. The target compound **42** is finally transformed under the same conditions as the first route.

**SCHEME 2:**
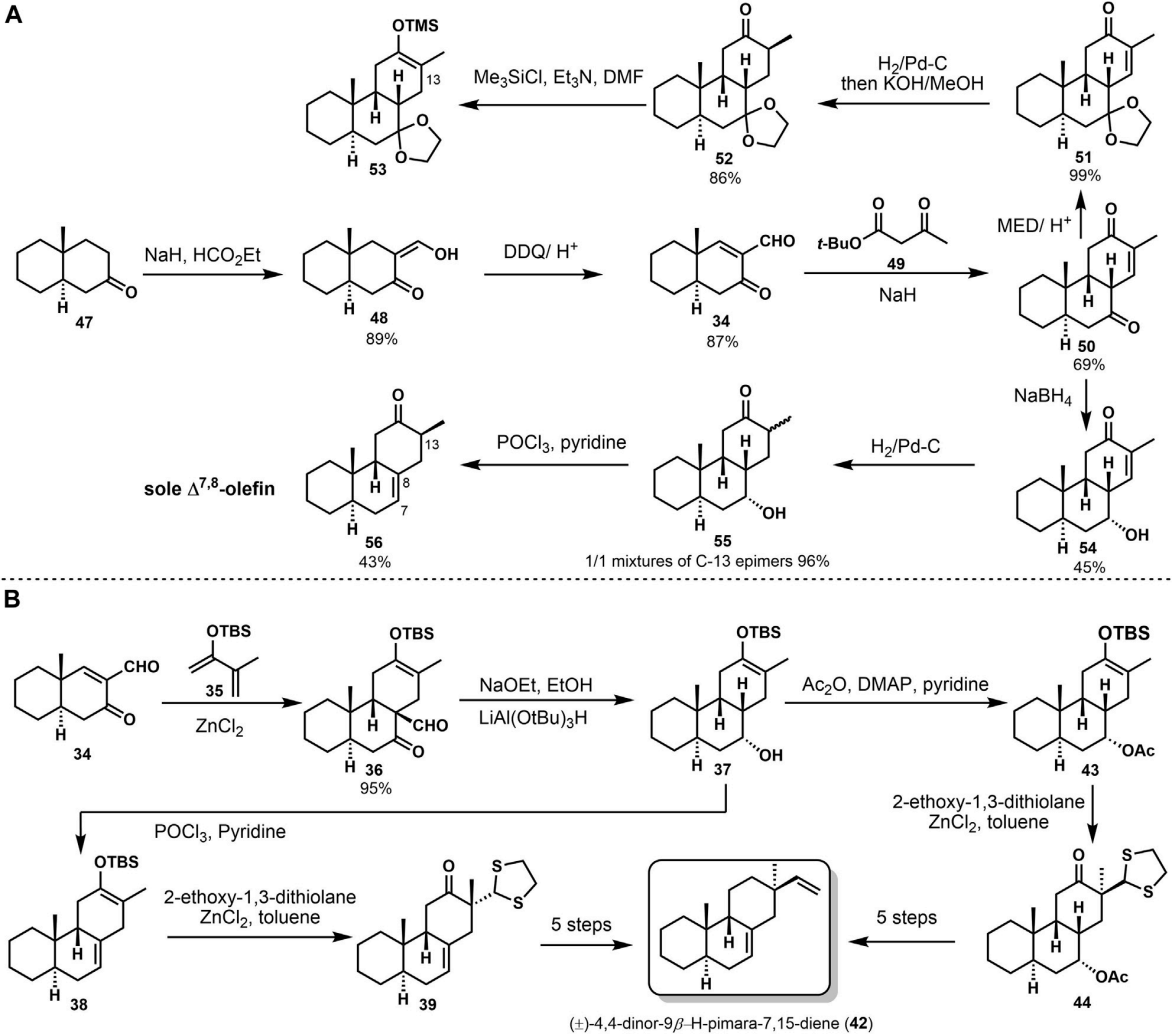
De Groot’s second synthesis of (±)-4,4-dinor-(9*β*-H)-pimara-7,15-diene in 1985.

**SCHEME 3:**
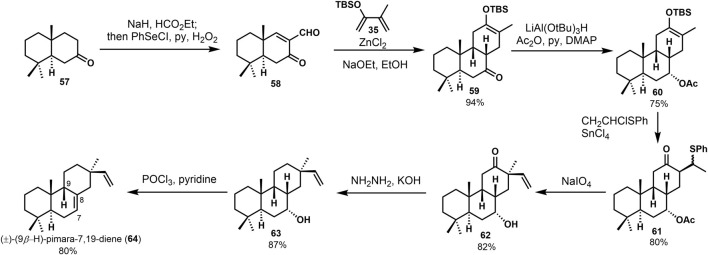
De Groot’s synthesis of (±)-(9*β*-H)-pimara-7,19-diene in 1989.

The stereoselective synthesis of (±)-4,4-dinor-9*β*-H-pimara-7,15-diene (**42**) was accomplished by Sicherer-Roetman Sicherer-Roetman et al. (1985). Initially Meyer (Meyer et al. 1975) formed the *trans-syn-cis* tricyclic product **50** with formyl enone **34** and tert-butyl 3-oxopentanoate **49** (Scheme 2A). Formylation and dehydrogenation of decalone **47** provided the starting compound **34**. To investigate the alkylation of **50** and get *β*-vinyl group at C-13, they prepared trimethylsilyl enol ether **53** after reduction, but product **53** would hydrolyze rapidly. Then they obtained **54** from 50 with the presence of NaBH_4_. Product **54** could be treated through hydrogenation and elimination to get **56**. Elimination of **55** only provided the Δ^7,8^-olefin in 43% yield. Another approach was based on the Diels–Alder reaction. They obtained regiospecific silyl enol ether **36** and provided the desired stereochemistry at C-9. Deformylation of product **36** and reduction with lithium tri-tert-butoxy aluminum hydride gave alcohol **37**. Then, product **37** was converted into model compound (±)-4,4-dinor-(9*β*-H)-pimara-7,15-diene (**42**) *via* several transformations (Scheme 2B). These conversions were reported in 1984 by Sicherer-Roetman (Sicherer-Roetman et al., 1984).

(±)-9*β*-H-pimara-7,19-diene (**64**) was seen as one of the intermediates in the biosynthesis of photoalexines in rice. It possessed the A, B, C ring system of momilactones. In 1989, Jansen (Jansen et al., 1989) reported the synthesis of (±)-9*β*-H-pimara-7,19-diene (**64**). They followed their previous syntheses to carry out a Diels–Alder reaction between enone aldehyde **57** and 2-(tert-butyldimethylsilyloxy)-3-methyl-1,3-butadiene **35**. Through deformylation and hydrogenation, with the hydroxyl group being protected, 7*α*-acetoxy compound **60** was provided. Stereoselective alkylation of the silyl enol ether **60** with CH_2_CHClSPh, followed by oxidation and elimination of the sulfoxide group, gained the desired vinyl product **62**. The carbonyl was removed during the Wolff–Kishner reduction of **62**. Finally, (±)-9*β*-H-pimara-7,19-diene (**64**) gave a 28% overall yield (Scheme 3).

**SCHEME 4:**
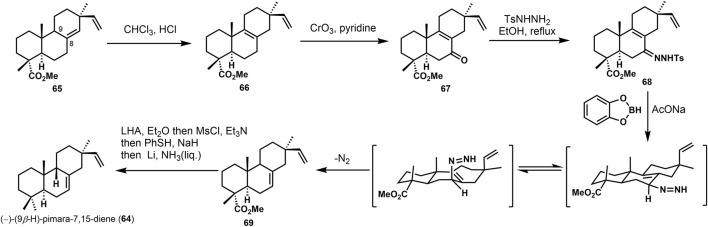
Coates’s synthesis of (−)-(9*β*-H)-pimara-7,15-diene in 1992.

The synthetic challenge of 9*β-*H pimarane skeleton could be to create the 9,10-*syn* configuration (Feilner et al., 2021). Several synthetic approaches have been accomplished (Feilner et al., 2020) to construct the stereochemistry at C-9,10 by Michael addition, lithium-ammonia reduction (Yu and Yu, 2015), and Diels–Alder reaction (Deslongchamps et al., 2014). Some of these strategies would gain the 9,10-*trans* products, inconsistent with the desired goal. In 1992, the 9,10-*syn* stereochemistry was accomplished via catechol borane reduction by Coates (Chu and Coates, 1992). As shown in Scheme 4, the unsaturated compound **66** was obtained from **65** by isomerization to its Δ^8^ isomer with HC1/CHC1_3_. Regioselective allylic oxidation of **66** provided ketene **67**. It was refluxed with *p*-toluene sulfonyl hydrazine in ethanol to obtain tosylhydrazone **68** and treated with catechol borane and sodium acetate. Double bond isomerization rearrangement was used, and Δ7,8-olefin **69** was obtained. Subsequently, the 4*α*-ester group of compound **69** was reduced by lithium aluminum-hydrogen to yield primary alcohol, and hydroxyl was protected after removing the methyl sulfonyl and fulguration. Finally, the target product (−)-(9*β*-H)-pimara-7,15-diene (**64**) was obtained by desulphurization with liquid lithium ammonia.

**SCHEME 5:**
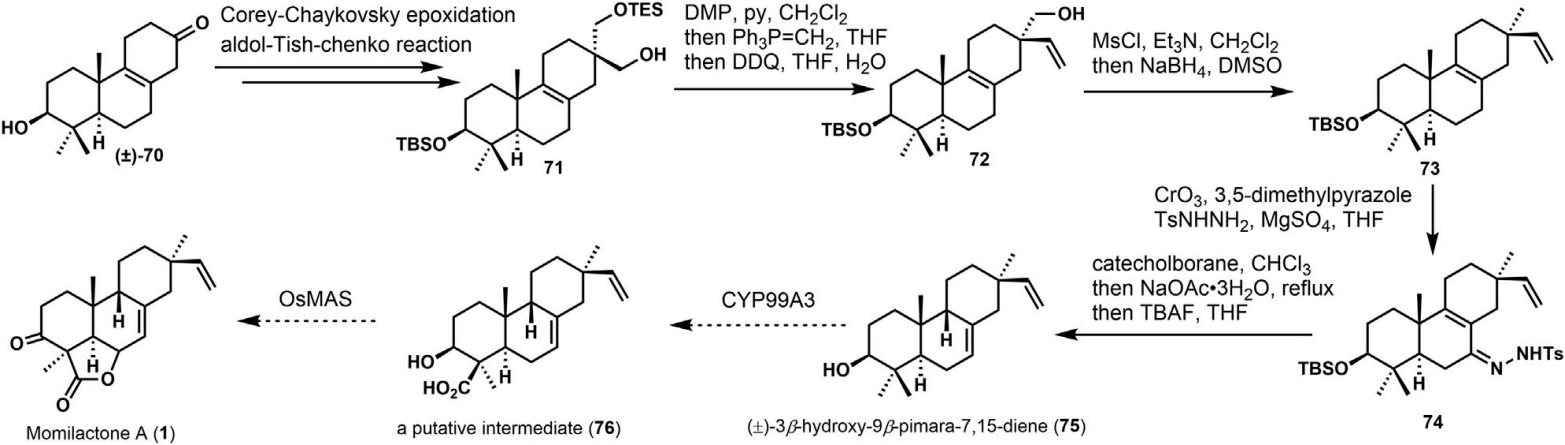
Yajiama’s synthesis of (±)-3*β*-hydroxy-9*β*-pimara-7,15-diene in 2011.

Yajiama (Yajiama et al., 2011) investigated the synthesis of (±)-3*β*-hydroxy-9*β*-pimara-7,15-diene (**75**). The core skeleton was constructed via Hutchins allyldiazene rearrangement (Chu and Coates, 1992). In Scheme 5, the approach started from the known ketone (±)−**70**, and **71** was gained *via* several transformations in good yield. Then, the hydroxyl group was oxidized. After the Witting olefination and deprotection, vinyl product **72** was obtained. The hydroxyl group of **72** was removed to get the desired derivative **73**. It possesses *β-*vinyl groups at C-13. After reducing **74** by catechol borane, under the presence of sodium acetate, the desired 9,10-*syn* tricyclic compound (±)−3*α*-hydroxy-9*β*-pimara-7,15-diene (**75**) was provided, which was considered a putative intermediate of momilactones and other diterpene phytoalexins in rice. It can be converted into **76** and momillactone A (**1**). In these syntheses, it furnished the configuration of the C-13 quaternary center using a stereoselective approach, and 9,10-*syn* tricyclic skeleton was constructed *via* rearrangement. This methodology would also apply to the synthesis of 9*β*-H pimaranes.

**SCHEME 6:**
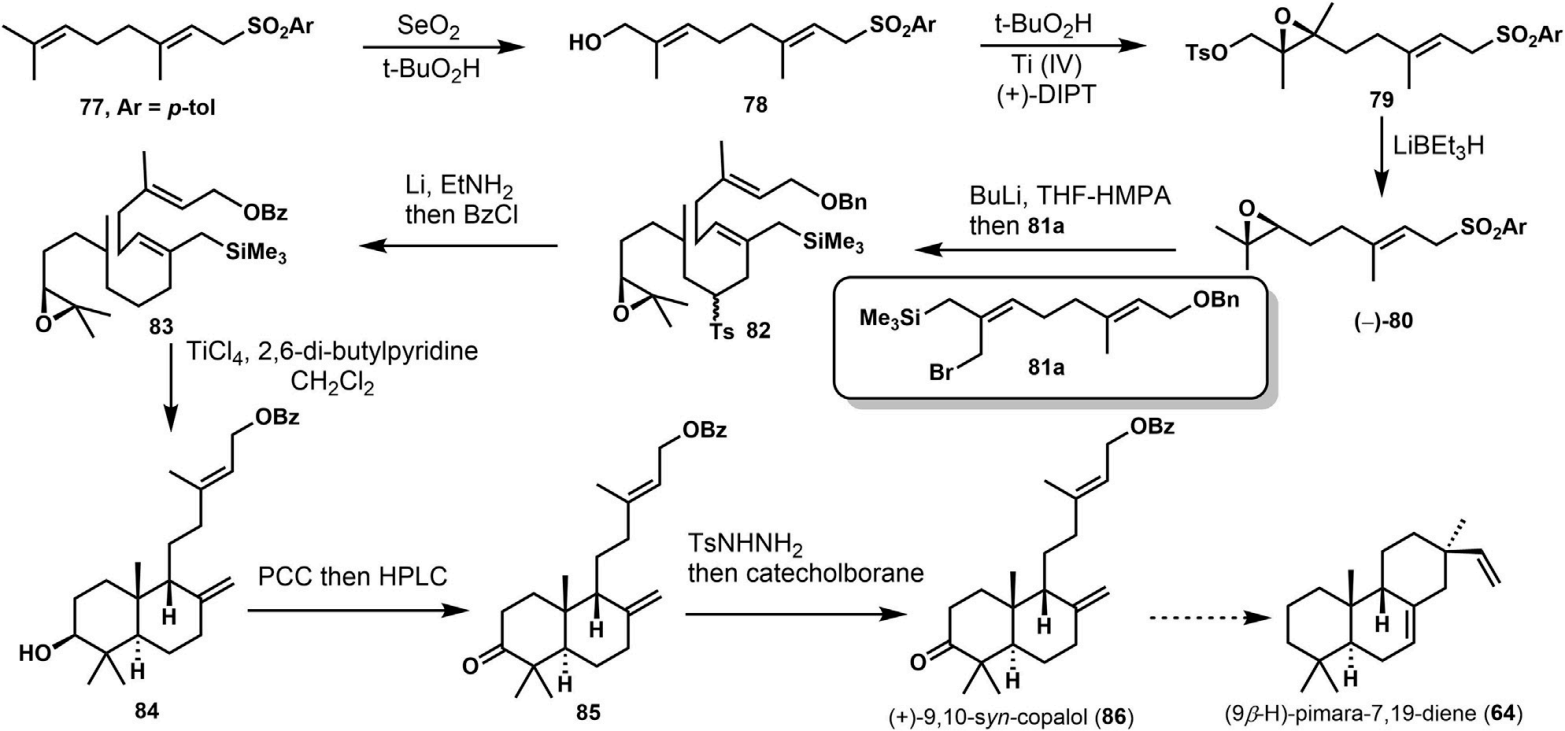
Coates’s synthesis of (+)-9,10-*syn*-copalol in 1992.

Yee and Coates (Yee and Coates, 1992) accomplished the synthesis of 9,10-*syn*-Copalol (**86**). In Scheme 6, the approach was started from **77** *via* Riley oxidation and Sharpless epoxidation under the presence of TiCl_4_. A conversion was performed to remove the hydroxyl group with LiBEt_3_H. Then, **82** was provided *via* lithiation and alkylation with (E, Z)-8-bromo-9-(trimethylsilyl) geranyl benzyl ethers (**81a**). Selective reductive cleavage of the toluenesulfonyl and protected benzyl group produced the tandem cyclization precursor **83**. Lewis acid treatment (TiCl_4_) of **83** afforded the stereorandom bicyclizations **84** and its diastereoisomers. Then, mixtures were oxidized and separated to get **85**. (+)-9,10-*syn*-copalol (**86**) was offered through the reduction with catecholborane. It could be converted to (9*β*-H)-pimaran-7,19-diene **64**) *via* another tandem cyclization.

**SCHEME 7:**
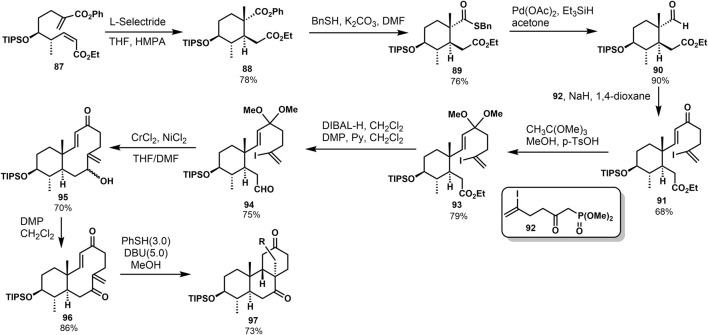
Nakada’s stereoselective approach to *tran-syn-cis* tricyclic system in 2014.

Fusidane triterpenes are a relatively small family of natural steroidal antibiotics, including fusidine, helvolic acid, and fusidic acid. These compounds have a unique chair-boat-chair ABC tricyclic ring system seen as a sort of 9*β*-pimara skeleton (Caron and Deslongchamps, 2010). In 2014, the intermolecular/transannular Michael reaction was first applied to the synthesis of ABC-ring in fusidane triterpenes by Fujii and Nakada (Fujii and Nakada, 2014). In Scheme 7, they developed the stereoselective intramolecular Michael reaction of compound **87** with L-Selectride to provide compound **88** (Scheme 7). Compound **88** was performed with benzyl thiol and potassium carbonate affording the benzyl thioester **89**. It was then converted to aldehyde **90** by Fukuyama reduction. Enone **91** was prepared *via* HWE reaction of aldehyde **90** and keto phosphonate **92**. The dimethyl acetal **93** was afforded from **91**, followed by reduction, and Dess–Martin oxidation gave aldehyde **94**. The intramolecular Cr-mediated reaction of compound **94** was optimized when the reaction was performed in THF/DMF mixture, offering sole product **95** (70%). After that, oxidation of compound **95** provided the *bis*-enone **96**, the substrate for intermolecular/transannular Michael reaction cascade. Then, they carried out the reaction of compound **96** under several conditions. Annulation product **97** was formed when thiophenol and DBU were used in methanol at 0 °C in a 73% yield.

**SCHEME 8:**
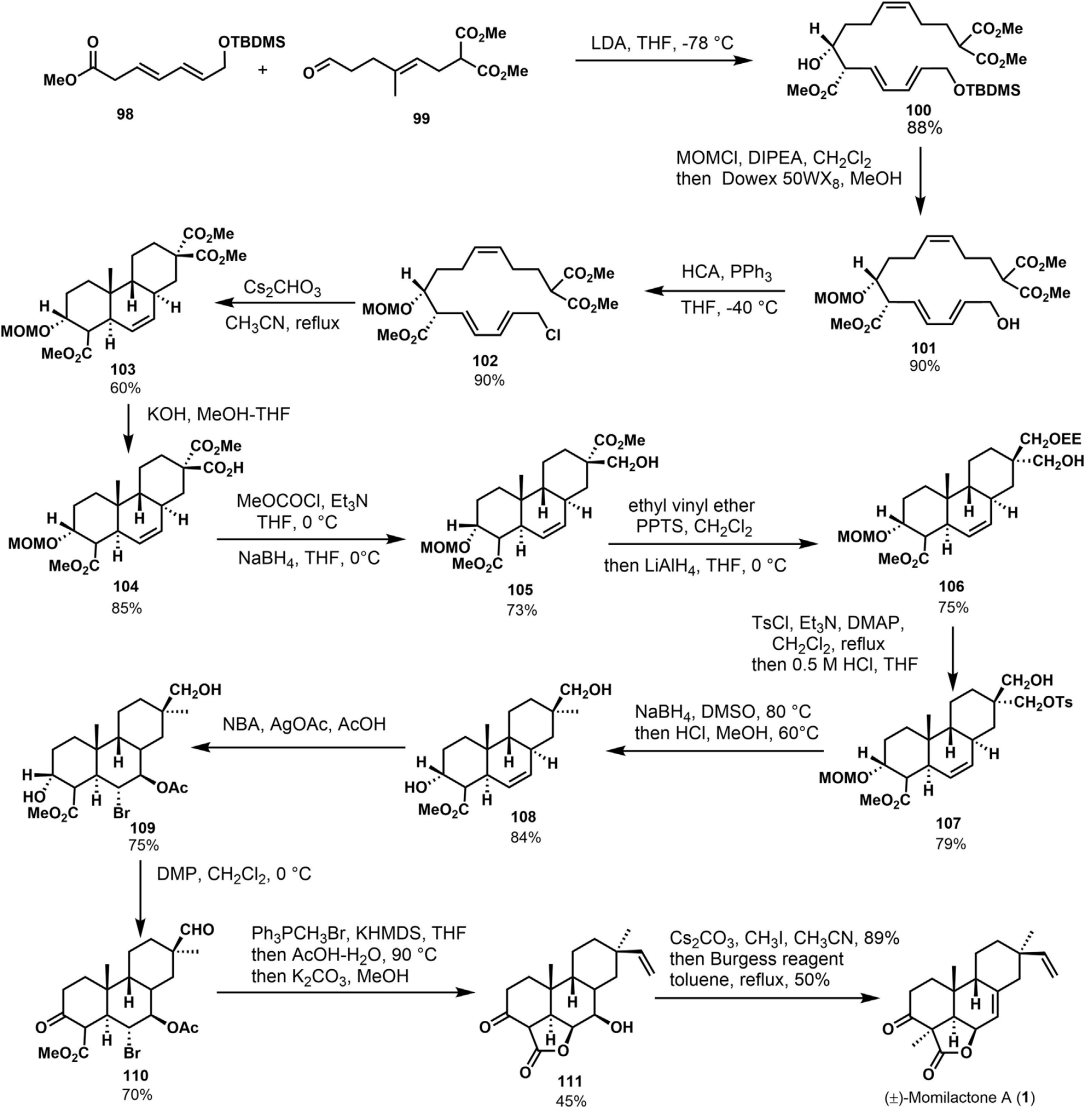
Deslongchamps’s total synthesis of (±)-momilactone A in 2002.

Germain and Deslongchamps (Germain and Deslongchamps, 2002) accomplished the first total synthesis of (±)-momilactone A (**1**) *via* a Diels–Alder reaction (Germain and Deslongchamps, 1999). Scheme 8 shows that the condensation was accomplished from conjugated olefins **98** and vinylaldehydes **99** with 88% yield to give diethylisomers **100**. Subsequently, MOM ether was obtained from **100** *via* the protection, followed by selective desilication of primary hydroxyl ether to obtain compound **102**. *Trans-syn-trans* tricyclic compound **103** was offered by Diels–Alder reaction with stereoselectivity under reflux in cesium carbonate acetonitrile solution. In a word, a series of conversions of **100** provided the diastereoisomer **103** in the chair-boat-chair configuration, which is consistent with (±)-momilactone A (**1**). The target product was obtained through linear strategy transformation starting from intermediate **103**. Malonate compound **103** underwent partial hydrolysis and several functional group transformations to afford intermediate **104**. Then, the double bond addition was performed under the action of NBS and silver acetate to obtain bromoacetate **109** with high stereoselectivity, followed by the Dess–Martin oxidation and Wittig reaction to obtain the alkenone. Under the condition of acetic acid-water, intramolecular esterification was performed. Moreover, the hydrolysis of acetyl ester was carried out to obtain hydroxylolactone **111**. Then, the target product (±)-momilactone A (**1**) was obtained by the carbonyl *α*-methylation and dehydration of lactone.

## Summary and Further Prospects

Some synthetic strategies have been reported about the construction of the 9*β*-H piamarane skeleton, such as Diels–Alder reaction, Michael addition, and catechol borane reduction. They carried out the syntheses of the skeleton and the intermediates in natural products using simple procedures. The asymmetric totals synthesis of 9*β*-H piamaranes has not been reported so far. A new approach must be applied to the natural products in 9*β*-H pimaranes.
